# Review of Hyperbaric Oxygen Therapy as an Adjunctive Intervention for Metabolic Disorders

**DOI:** 10.3390/antiox14121443

**Published:** 2025-11-30

**Authors:** Renata Karaś, Urszula E. Binduga, Paweł Januszewicz, Konrad A. Szychowski

**Affiliations:** 1 Renata Karaś’s Dietetic Office, Podole 145A, 39-320 Przecław, Poland; renata35b@o2.pl; 2 Department of Lifestyle Disorders and Regenerative Medicine, Medical College, University of Information Technology and Management in Rzeszow, Sucharskiego 2, 35-225 Rzeszow, Poland; ubinduga@wsiz.edu.pl (U.E.B.); pjanuszewicz@wsiz.edu.pl (P.J.); 3 Department of Biotechnology and Cell Biology, Medical College, University of Information Technology and Management in Rzeszow, st. Sucharskiego 2, 35-225 Rzeszow, Poland

**Keywords:** hyperbaric oxygen therapy, obesity, insulin resistance

## Abstract

Obesity is a chronic systemic disease characterised by insulin resistance, inflammation, and mitochondrial dysfunction. Hyperbaric oxygen therapy (HBOT), which involves the administration of 100% oxygen under elevated atmospheric pressure, has a well-established clinical application in the treatment of non-healing wounds and ischemia, and it is currently being investigated as an adjunctive therapy for obesity and metabolic disorders. The aim of this review is to provide a critical synthesis of recent (2012–2025) evidence regarding the mechanisms of HBOT action in the human body. Furthermore, it examines the metabolic effects and safety profile of HBOT in the context of obesity, with particular attention to experimental and preliminary clinical research. Preclinical studies have demonstrated that HBOT enhances insulin sensitivity, reduces adipose tissue inflammation, and modulates lipid metabolism. The proposed mechanisms include activation of Akt/AMPK signalling and GLUT4 translocation in skeletal muscle, resulting in improved glucose uptake and oxidation, as well as stimulation of thermogenesis in brown adipose tissue. In rodent models of obesity, HBOT has been shown to reduce adipose tissue mass, improve lipid profiles, and restore normal β-oxidation of fatty acids by normalising the expression of peroxisome proliferator-activated receptor-α and carnitine palmitoyl transferase 1B in muscle tissue. Preliminary clinical studies in humans indicate that HBOT enhances both systemic and tissue insulin sensitivity, accompanied by improved mitochondrial function and reduced endoplasmic reticulum stress. Despite these promising findings, data on the long-term efficacy, optimal treatment protocols, and safety of HBOT in obese individuals remain limited. In conclusion, HBOT appears to be a promising adjunctive approach in the management of obesity through the multidirectional improvement in metabolic functions. However, high-quality clinical trials are required to confirm its effectiveness, durability of outcomes, and safety profile across diverse patient populations.

## 1. Introduction

Obesity is a global health problem characterised by excessive accumulation of adipose tissue and a chronic low-grade inflammatory state, which contributes to the development of insulin resistance and metabolic disorders [1,2,3]. In obesity, adipose tissue shows increased infiltration of immune cells and heightened release of pro-inflammatory mediators. Consequently, insulin secretion becomes impaired, leading to the onset of insulin resistance [4,5]. The inflammatory process within adipose tissue represents a key mechanism linking obesity with peripheral insulin resistance, type 2 diabetes mellitus (T2DM), and cardiovascular disease [1,6]. Conventional strategies for managing obesity, including lifestyle modification, pharmacotherapy, and bariatric surgery, demonstrate variable efficacy and are associated with several limitations, thereby justifying the growing interest in novel adjunctive interventions [7,8].

Hyperbaric oxygen therapy (HBOT) involves breathing 100% oxygen at pressures typically ranging from 2.0 to 2.8 atmospheres absolute (ATA) for 60–120 min within a hyperbaric chamber [9]. By increasing ambient pressure, HBOT elevates the amount of oxygen dissolved in plasma and enhances tissue oxygenation by up to approximately fourfold [10]. HBOT is a well-established therapeutic approach for conditions such as decompression sickness, carbon monoxide (CO) poisoning, chronic non-healing wounds, and radiation-induced tissue injury [11,12,13,14]. This broad clinical utility stems from its capacity to improve oxygen delivery, stimulate angiogenesis, and modulate inflammatory responses [15].

Because of its non-invasive, painless, and relatively safe nature, HBOT has been widely implemented in clinical practice [16]. Repeated HBOT sessions are commonly used in patients with diabetes to support the healing of diabetic foot ulcers [13,17]. Moreover, even a single session has been reported to induce a rapid reduction in blood glucose levels [18]. These observations suggest that HBOT may exert notable effects on systemic metabolism.

The aim of this review is to synthesise and critically evaluate contemporary evidence (2012–2025) concerning the use of HBOT as an adjunctive therapy for obesity and metabolic disorders, and to summarise current data on its mechanisms of action, efficacy, and safety profile.

## 2. Materials and Methods

A systematic literature search was conducted in the PubMed, Scopus, and Web of Science databases for studies published between January 2012 and July 2025. The search strategy included the following keywords and their combinations: “hyperbaric oxygen therapy”, “metabolic disorders”, “insulin resistance”, “inflammation,” and “metabolic syndrome”. The inclusion criteria comprised full-text, English-language original research articles involving human or animal models relevant to the topic, as well as clinical studies. Review articles and case reports were excluded, with the exception of a small number of clinically justified cases explicitly noted in the manuscript.

## 3. Mechanisms of HBOT Action in Metabolic Regulation

### 3.1. Improvement in Insulin Sensitivity and Glucose Uptake

In recent years, there has been growing interest in the use of HBOT for managing insulin resistance and T2DM [19,20]. Wilkinson et al. (2020) demonstrated that HBOT significantly improves insulin sensitivity in men with T2DM, whereas exposure to hyperbaric air does not produce a comparable effect [20]. The underlying mechanism is likely related to direct stimulation of metabolic processes through enhanced oxygen availability or the induction of mild oxidative stress [21]. In a study involving men with T2DM (n = 12), a single HBOT session improved insulin sensitivity in both adipose and skeletal muscle tissues, increased phosphorylation of insulin receptor substrate 1 (IRS-1) and activation of protein kinase B (Akt), and simultaneously reduced endoplasmic reticulum (ER) stress [10]. At the same time, mitochondrial oxidative capacity was improved as indicated by an elevated reduced-to-oxidised glutathione ratio, suggesting that mitohormetic mechanisms may contribute to the observed metabolic effects [10].

At the molecular level, the metabolic effects of HBOT arise from activation of key signalling pathways involved in glucose uptake and substrate oxidation [10,22]. Exposure to elevated partial pressure of oxygen transiently increases oxidative stress, which subsequently activates adenosine monophosphate-activated protein kinase (AMPK) and the IRS-1/Akt signalling cascade. This leads to greater translocation of glucose transporter type 4 (GLUT4) to the cell membrane and enhanced glucose uptake in skeletal muscle [10,23]. Concurrently, HBOT stimulates the expression of transcriptional regulators involved in mitochondrial biogenesis, such as sirtuin 1 (SIRT1) and peroxisome proliferator-activated receptor gamma coactivator 1-alpha (PGC-1α), thereby enhancing cellular metabolic activity and oxidative capacity [24].

In adipocytes, HBOT has been shown to increase adiponectin levels and suppress the expression of resistin and plasminogen activator inhibitor-1, possibly through activation of the peroxisome proliferator-activated receptor gamma (PPARγ)–adiponectin axis [25]. These effects are further supported by improved nitric oxide (NO) signalling, which facilitates microcirculatory perfusion in peripheral tissues [26]. Parallel experimental studies in murine and rat models confirm the beneficial impact of HBOT on metabolic homeostasis [25,27,28,29]. It has been demonstrated that short-term HBOT in diabetic mice reduces fasting glycaemia, improves insulin sensitivity as assessed by the HOMA-IR index, and promotes regeneration and proliferation of pancreatic β-cells via modulation of the Bcl-2/caspase-3/poly(ADP-ribose) polymerase pathway, thereby inhibiting apoptosis [19]. Additionally, HBOT reduces hepatocellular injury and increases hepatic glycogen storage [19]. In this model, a decline in food intake was also observed in HBOT-treated mice, suggesting a possible influence of the therapy on hypothalamic appetite-regulating centres.

Recent studies further support these mechanistic observations. Xie et al. (2025) demonstrated that exposure to HBOT effectively suppresses diet-induced hyperphagia in high-fat-fed mice by increasing levels of the anorexigenic peptide nesfatin-1 in both the hypothalamus and peripheral tissues [30]. The HBOT-induced elevation of nesfatin-1 was accompanied by normalisation of aberrant neuronal activation in the arcuate and paraventricular nuclei of the hypothalamus. Pharmacological blockade of the melanocortin-3/4 receptor pathway abolished the beneficial effects of HBOT on food intake, visceral adipose tissue mass, and body weight gain [30]. Emerging data also suggest that anti-inflammatory and redox-regulatory mechanisms play a significant role in the metabolic action of HBOT. The therapy inhibits activation of the nucleotide-binding oligomerisation domain (NOD)-like receptor family pyrin domain-containing protein 3 (NLRP3) inflammasome, thereby reducing the release of interleukin (IL)-1β and tumour necrosis factor-α (TNF-α) in metabolic tissues. Moreover, it promotes macrophage polarisation from the pro-inflammatory M1 phenotype towards the reparative M2 phenotype, thus attenuating local inflammation [31,32]. In other experimental models using Otsuka Long-Evans Tokushima Fatty (OLETF) and Long-Evans Tokushima Otsuka (LETO) rats, HBOT improved glucose tolerance by increasing the activity of mitochondrial oxidative enzymes such as cytochrome c oxidase, succinate dehydrogenase, and β-hydroxyacyl-CoA dehydrogenase, indicating an enhancement of skeletal muscle oxidative capacity [33]. The authors suggested that HBOT counteracts diabetes-related alterations in muscle fibre composition, particularly the loss of oxidative type I fibres in favour of glycolytic type IIb fibres [33]. In murine models of T2DM, HBOT has also been shown to increase GLUT4 translocation and AMPK activation in skeletal muscle, as well as to enhance fatty acid oxidation and the expression of uncoupling protein 1 (UCP1) in adipocytes, thereby promoting browning of white adipose tissue (WAT) and improving overall energy metabolism [23]. Furthermore, HBOT may contribute to increased energy expenditure, a potential mechanism underlying its anti-obesity effects [23].

PPARs constitute a family of nuclear transcription factors that play a central role in maintaining metabolic homeostasis [34]. They regulate the expression of genes involved in lipid and glucose metabolism, inflammation, and mitochondrial function within insulin-sensitive tissues such as adipose tissue, skeletal muscle, and the liver [35,36]. Among these isoforms, PPARγ is highly expressed in adipocytes, where it governs adipocyte differentiation, adipokine expression (including adiponectin and leptin), lipid storage, and metabolic crosstalk between adipose tissue and other organs. Activation of PPARγ represents a key mechanism underlying improved insulin sensitivity and glucose regulation. By promoting fatty acid uptake and storage within adipocytes, PPARγ activation reduces lipotoxicity and prevents ectopic lipid accumulation in non-adipose tissues [37].

From the perspective of lipid regulation, HBOT influences the activation of PPARα and PPARδ, which enhance β-oxidation of fatty acids in skeletal muscle and liver, thereby improving the lipid profile [34,38,39]. Consequently, HBOT supports not only more efficient carbohydrate metabolism but also intensified fat oxidation, leading to an overall improvement in metabolic performance [35,39].

In a study by Quintero et al. (2012), the effects of hyperoxia (95% O_2_ for 24 h) were examined in differentiated 3T3-L1 adipocytes to assess the cellular response of adipose tissue to elevated oxygen availability [40]. The results revealed a marked increase in reactive oxygen species (ROS) production, accompanied by decreased glucose uptake and lactate secretion, alongside elevated glycerol release, indicating a metabolic shift towards lipolysis [40]. At the transcriptional level, there was a pronounced upregulation of PPARγ, IL-6, and monocyte chemoattractant protein-1, together with a reduction in angiopoietin-like 4 expression, confirming that hyperoxia modulates key regulators of lipid metabolism and inflammatory responses [40]. These findings suggest that short-term exposure to hyperoxia elicits an adaptive oxidative stress response capable of modulating crucial signalling pathways in adipocytes.

Collectively, these results underscore the significant contribution of anti-inflammatory and redox-dependent mechanisms to the metabolic effects of HBOT.

### 3.2. Impact of HBOT on Endothelial Barrier Integrity

In obesity and diabetes, endothelial dysfunction manifests as loss of barrier integrity and heightened inflammatory adhesion [41]. In vitro and in vivo studies indicate that HBOT can attenuate these derangements. For example, hyperoxia reduces endothelial–leukocyte adhesion and downstream cytokine release, thereby limiting oedema and preserving vascular integrity [42]. In human umbilical vein endothelial cells (HUVEC) exposed to hypoxia with inflammatory stimuli such as lipopolysaccharide (LPS) and TNF-α, HBO reversed neutrophil adhesion by downregulating endothelial adhesion molecules such as intercellular adhesion molecule 1 (ICAM-1) and vascular cell adhesion molecule-1 (VCAM-1) [43]. This effect was linked to increased S-nitrosation under HBOT, suggesting that reactive nitrogen signalling helps stabilise junctional proteins and prevent barrier leak [44]. Thus, HBOT anti-inflammatory impact on endothelium via reduced adhesion molecule expression and leukocyte binding, likely improves barrier function in metabolic vascular beds [42,43].

Moreover, HBOT robustly stimulates endothelial proliferation and new vessel growth in metabolic disease models. In rodent diabetic-wound and ischemia models, repeated HBOT exposures increased capillary density and endothelial cell number in injured tissue [28]. Mechanistically, HBOT upregulates hypoxia-responsive angiogenic pathways despite the high oxygen milieu [45]. Has been described that in diabetic mice treated with HBOT showed elevated hypoxia-inducible factor 1-alpha (HIF-1α) and downstream factors such as vascular endothelial growth factor A (VEGF-A) and, stromal cell-derived factor 1 (SDF-1) as well as endothelial VEGF receptors and C-X-C chemokine receptor type 4 (CXCR4) [45,46]. In vitro, HBOT restored endothelial proliferation, migration and tube formation in HUVEC challenged with high glucose and oxygen–glucose deprivation [47]. Likewise, HBOT increased expression of angiogenic proteins and sirtuin-1 (SIRT1) in obese or ischemic models, while reducing apoptosis mediators [48]. These findings demonstrate that HBO drives compensatory neovascularization in metabolic tissues by enhancing endothelial angiogenic signalling.

HBOT also modulates endothelium-derived vasoactive mediators in complex ways, taking into due consideration the structure and functional integrity of the endothelial glycocalyx [49]. It tends to elevate pro-angiogenic and vasodilatory signals such as VEGF/NO pathways, while variably affecting constrictors [45]. In diabetic or wound contexts, HBOT induces VEGF and SDF-1 release by stromal and endothelial cells, supporting vasodilation and angiogenesis [45]. However, in patients with peripheral vascular disease, HBOT paradoxically increased endothelin 1 (ET-1) levels which is a potent vasoconstrictor [50]. Indeed, in a clinical HBOT regimen for PAD, plasma ET-1 rose significantly (from ~4 to 18 pg/mL) despite little change in NO [50]. Thus, although HBOT boosts angiogenic mediators, it can also shift the vasoactive balance toward constriction in some disease states. The effect in obesity-related vasculopathy may depend on context. Therefore, HBOT can improving microvascular perfusion by angiogenesis while simultaneously modulating systemic vasomotor tone via altered ET-1/NO release.

Finally, HBOT consistently dampens endothelial–leukocyte crosstalk in metabolic inflammation. In metabolically activated endothelium, upregulation of ICAM-1 and VCAM-1 promotes firm leukocyte adhesion, transmigration, and release of oxidative and proteolytic mediators that exacerbate vascular injury. HBOT counteracts this pro-adhesive phenotype [43]. Similar suppression of ICAM-1 was observed in hypoxia–hypoglycaemia models, where HBOT reduced ICAM-1 mRNA and protein and limited neutrophil binding via NOS-regulated pathways [44]. HBOT also alters human leukocyte behaviour directly. Brief hyperoxic exposure transiently downregulates β2-integrin (Mac-1) activity in neutrophils, reducing their ability to anchor to activated HUVEC [51]. Additional microvascular studies demonstrate that HBOT inhibits leukocyte rolling and adhesion in pulmonary and systemic vessels, further confirming its broad anti-inflammatory effects on the endothelium–leukocyte interface [52]. Clinical data likewise indicate that HBOT reduces adhesive integrin activation (α4β1, β2) on circulating neutrophils in patients with chronic inflammatory wounds [53]. Reduced leukocyte binding translates into less oxidative and proteolytic stress imposed on the vascular wall, preserving endothelial junctions and limiting microvascular injury. In a murine model of autologous fat grafting, short-course HBOT significantly decreased inflammatory oedema, reduced leukocyte infiltration, and maintained microvascular architecture, indicating a direct protective effect on inflamed tissue [42].

Together with the barrier-stabilising effects described above, these findings demonstrate that HBOT interrupts the self-reinforcing cycle of endothelial activation, leukocyte adhesion, and extravasation that drives obesity-related vascular inflammation. By simultaneously reducing endothelial adhesivity, suppressing leukocyte recruitment, and limiting inflammatory effector damage, HBOT restores microvascular homeostasis in metabolically compromised tissues.

### 3.3. Effects on Adipose Tissue, Lipid Metabolism, and Energy Expenditure

In mammals, three principal types of adipose tissue are distinguished: WAT, brown adipose tissue (BAT), and beige adipose tissue [24]. WAT serves primarily as an energy reservoir [54]. It accumulates predominantly in the visceral region, thereby increasing the risk of metabolic disorders [24]. By contrast, BAT performs a distinct physiological function—its main role is non-shivering thermogenesis, i.e., the production of heat through metabolic processes [55,56]. Moreover, BAT has been shown to contribute to the reduction in circulating glucose and triglyceride levels, thereby ameliorating manifestations of the metabolic syndrome [56]. Beige adipose tissue, regarded as an intermediate form between WAT and BAT, is characterised by a high mitochondrial content and the presence of UCP1 [24]. These cells, interspersed within WAT depots, play an important role in regulating energy balance and thermogenic capacity [24,57].

In recent years, accumulating evidence has indicated that HBOT can modulate adipose tissue function and promote thermogenic processes [23,24,28]. In rat studies, exposure to HBOT increased BAT volume, enhanced glucose uptake, and upregulated key regulators of thermogenesis such as UCP1 and PGC-1α [24]. These changes were accompanied by improved lipid metabolism, suggesting that HBOT may contribute to the reduction in excessive metabolic reserves [24]. Similar effects have been observed in mice with T2DM, where HBOT induced the browning of WAT, increasing the expression of UCP1 and GLUT4 and activating AMPK [23]. In obese and diabetic rat models, HBOT improved insulin sensitivity and glucose tolerance, while promoting the browning of WAT and generating adipocytes with thermogenic properties [58]. Yin et al. (2024) demonstrated that HBOT can stimulate endothelial cells to release exosomes, which subsequently promote UCP1 expression and the conversion of white adipocytes into cells with BAT-like characteristics [28]. These findings indicate that the beneficial metabolic effects of HBOT may be partly mediated through intercellular communication between the vascular endothelium and adipocytes. Exosomes derived from endothelial cells exposed to HBOT were shown to trigger thermogenic programmes in adipocytes, thereby increasing energy expenditure and improving glucose–lipid homeostasis [28].

Furthermore, in models of metabolic syndrome, HBOT reduced body weight, decreased visceral adipocyte hypertrophy, and normalised lipid profiles [27]. Notably, fasting glucose and insulin concentrations decreased, HOMA-IR values were reduced, and markers of dyslipidaemia and non-alcoholic fatty liver disease—such as alanine aminotransferase and aspartate aminotransferase—were normalised [27]. In addition, insulin sensitivity indices including 1/HOMA-IR, HOMA-β, and the McAuley index were significantly improved, confirming that the enhanced metabolic performance was linked to improved β-cell function and systemic insulin responsiveness [27].

In a study employing apolipoprotein E-deficient (ApoE^−^/^−^) mice, a classical model of lipid dysregulation, compelling evidence was also provided for the neuroprotective effects of HBOT [38]. HBOT-treated animals exhibited improved cognitive performance in behavioural tests, substantial normalisation of lipid profiles, and a reduction in body weight. In the hippocampus, decreased β-amyloid deposition and preservation of neuronal integrity were observed [38]. These neuroprotective outcomes were associated with reductions in oxidative stress markers and inflammatory mediators in both serum and brain tissue. Collectively, these findings indicate that HBOT simultaneously influences lipid metabolism and the neurovascular microenvironment, underscoring its multidimensional systemic effects [38].

From a molecular perspective, particular attention should be given to the PPARs—specifically the α, γ, and δ isoforms—which play pivotal roles in regulating lipid metabolism and energy homeostasis [34,35,39]. PPARα primarily enhances β-oxidation of fatty acids in the liver and skeletal muscle by upregulating enzymes involved in lipid catabolism. PPARγ, by contrast, governs adipocyte differentiation and triglyceride storage while simultaneously increasing the insulin sensitivity of adipose cells [35,39,59]. PPARδ (also referred to as PPARβ/δ) promotes fatty acid oxidation and energy expenditure in peripheral tissues; activation of this receptor is associated with the induction of WAT browning and improvement in the lipid profile [36,39,60]. It is therefore plausible that the multidirectional metabolic effects of HBOT on lipid metabolism and adipocyte function are mediated through modulation of all three PPAR isoforms. Such modulation could enhance fatty acid oxidation via the PPARα–carnitine palmitoyltransferase 1 (CPT1) axis, while simultaneously reprogramming adipocytes towards a brown-like thermogenic phenotype [61]. Activation of this pathway would contribute to the reduction in excessive energy stores and improvement in systemic insulin sensitivity.

Recent findings also suggest that the influence of HBOT on lipid metabolism may extend to the direct regulation of fatty acid composition and enzymatic activity involved in lipid oxidation [62]. Resanović et al. demonstrated that in patients with type 1 diabetes mellitus, 10 HBOT sessions resulted in significant alterations in plasma fatty acid composition, including an increased n-3/n-6 polyunsaturated fatty acid ratio and a reduction in saturated fatty acids. These changes favourably modulated the lipid profile and reduced oxidative stress [62]. Collectively, these results indicate that HBOT may indirectly potentiate β-oxidation pathways by modifying lipid substrates and improving the cellular redox environment.

### 3.4. Oxidative Stress, Mitochondrial Function, and Inflammation

Dysfunction of adipose tissue plays a pivotal role in the development of metabolic disturbances associated with obesity and T2DM [63]. Under conditions of excessive fat accumulation, adipocytes enlarge, lose their physiological endocrine function, and initiate inflammatory and fibrotic processes within the tissue [64,65]. These alterations contribute to lipotoxicity and insulin resistance [65,66]. Among the most critical regulators of adipocyte function are mitochondria [67]. Initially, oxidative stress induced by a high-fat diet (HFD) is counterbalanced by a physiological process known as mitohormesis—an adaptive cellular response to transient oxidative stress [65]. However, with chronic high-fat feeding, adipocytes exhibit mitochondrial fragmentation and altered mitochondrial dynamics, processes associated with impaired mitohormetic adaptation [67,68]. In humans, even a single session of HBOT has been shown to enhance insulin sensitivity in the liver, skeletal muscle, and WAT of individuals with a body mass index of >30 kg/m^2^. These effects were attributed to the induction of mitohormesis and modulation of pro- and anti-inflammatory cytokine profiles [10]. In skeletal muscle, improved insulin sensitivity was associated with increased phosphorylation of Akt and decreased serine phosphorylation of IRS-1 [10].

In db/db diabetic mouse models, HBOT significantly alleviated renal injury [69]. The authors reported reduced urinary levels of kidney damage markers and decreased activity of caspase-3 in renal tissue [69]. HBOT also lowered proteinuria and modulated the expression of stress-response genes such as nuclear factor erythroid 2-related factor 2 (*NRF2*) and haem oxygenase 1 (*HMOX1*). The observed effects were attributed to stimulation of endogenous antioxidant pathways triggered by a moderate increase in oxygen availability. HBOT induced a mild, non-damaging rise in ROS, which activated cytoprotective signalling mechanisms—particularly the NRF2 pathway and heat shock proteins—ultimately suppressing excessive inflammation-associated ROS generation [69]. This sequence enhances the cellular antioxidant potential and mitigates chronic inflammation.

In mice fed a HFD, HBOT reduced lipid accumulation within adipocytes and restored balance in fatty acid metabolism [61]. Upregulation of PPARα and hormone-sensitive lipase was observed, along with modulation of CPT1B expression, indicating a beneficial effect on β-oxidation and mobilisation of stored lipids [61]. In BAT, HBOT suppressed the diet-induced overexpression of UCP1, the key thermogenic protein, suggesting a stabilising effect on the balance between energy storage and dissipation [61]. Beyond its metabolic effects, HBOT supports wound healing through complex regulation of oxidative stress, inflammation, angiogenesis, and extracellular matrix remodelling. The therapy reduces cellular damage markers, including creatine phosphokinase and aspartate aminotransferase. Although HBOT initially increases ROS production, it subsequently enhances the expression of antioxidant enzymes such as catalase and superoxide dismutase, while reducing the activity of xanthine oxidase and myeloperoxidase. This shift decreases oxidative damage markers such as malondialdehyde (MDA) [70]. HBOT also downregulates pro-inflammatory cytokines including TNF-α, IL-1β, and IL-6, while upregulating the anti-inflammatory cytokine IL-10. The mechanism involves inhibition of the nuclear factor kappa B pathway via stabilisation of its inhibitory subunit IκBα, thereby limiting the transcription of pro-inflammatory genes [70]. At the gene expression level, HBOT activates the transcription factors NRF2 and HIF-1α, which coordinate antioxidant and hypoxic responses, respectively. HIF-1α further stimulates the expression of growth factors—vascular endothelial growth factor, transforming growth factor-β, and platelet-derived growth factor—contributing to tissue repair and re-epithelialisation [70].

Similar protective mechanisms have been observed in other organ systems. In a rat model of obesity and accelerated ageing, HBOT reduced myocardial expression of the pro-inflammatory cytokine TNF-α and decreased the number of apoptotic cardiomyocytes, while simultaneously improving mitochondrial function within the heart [71]. The authors also reported lower mitochondrial ROS production, improved membrane potential, and reduced mitochondrial swelling. As a result, treated animals exhibited restoration of normal left ventricular ejection fraction, whereas untreated rats developed systolic dysfunction [71]. These findings highlight the potential of HBOT as a cardioprotective intervention in the context of metabolic syndrome, exerting anti-inflammatory, anti-apoptotic, and mitochondria-stabilising effects in cardiac tissue. Studies in neurodegenerative disease models have similarly demonstrated that HBOT enhances the expression of SIRT1 and PGC-1α, stimulates mitochondrial biogenesis, and protects neurons from apoptosis [72,73].

Sirtuins (SIRT1–SIRT7) are NAD^+^-dependent deacetylases involved in cellular energy homeostasis, apoptosis, and ageing processes [74]. Activation of SIRT1 increases the expression and activity of PGC-1α, which serves as the master regulator of mitochondrial biogenesis and functions as a co-activator of transcription factors such as mitochondrial transcription factor A (TFAM) that drive mitochondrial DNA replication and organelle formation [72]. In neurodegenerative models, activation of PGC-1α protects neurons from oxidative stress and apoptosis, underscoring its importance in maintaining mitochondrial integrity [72]. Additionally, voltage-dependent anion-selective channel proteins, located in the outer mitochondrial membrane, are involved in the regulation of metabolism and apoptosis [72]. In murine models, HBOT has been shown to potentiate this signalling pathway by upregulating the expression of SIRT1, PGC-1α, TFAM, and voltage-dependent anion-selective channel proteins, confirming its ability to induce mitochondrial biogenesis and enhance mitochondrial functionality [72]. These results suggest that the beneficial metabolic effects of HBOT may be mediated through SIRT1/PGC-1α-dependent mechanisms [72].

HBOT also exerts significant effects in other metabolically active organs. In rats with blunt liver injury, HBOT markedly accelerated hepatic regeneration and limited tissue damage [16]. The levels of the antioxidant enzyme superoxide dismutase remained significantly higher in the HBOT-treated group than in controls, indicating preservation of hepatic antioxidant capacity [16]. Conversely, levels of the lipid peroxidation marker MDA were markedly lower in HBOT-treated animals at critical post-injury time points [16]. In parallel, HBOT suppressed the rise in pro-inflammatory cytokines IL-1β, IL-6, and TNF-α in both hepatic tissue and serum [16].

Observations on the metabolic effects of HBOT are not limited to experimental models. In a study conducted on athletes, HBOT significantly increased maximal oxygen uptake and shifted the anaerobic threshold, reflecting enhanced mitochondrial performance in skeletal muscle [75]. One of the primary pathways governing mitochondrial biogenesis involves the SIRT1–PGC-1α–TFAM signalling cascade [76]. SIRT1 acts as a metabolic sensor, deacetylating PGC-1α and thereby activating the transcriptional programme responsible for mitochondrial proliferation [76]. HBOT has been shown to potentiate this cascade. In murine neurodegenerative models, therapy increased the expression of SIRT1, PGC-1α, and TFAM, leading to a marked rise in mitochondrial number and function [72]. A similar mechanism may operate in metabolically active tissues of obese individuals undergoing HBOT, where it could trigger mitohormesis, fostering mitochondrial recovery and enhancing cellular aerobic performance. This adaptive response supports the restoration of mitochondrial efficiency and improved systemic energy homeostasis. Modulation of oxidative stress by HBOT does not rely solely on suppression of excessive ROS production; it also involves adaptive stimulation of mitochondrial activity. In a study by de Wolde et al. involving healthy volunteers, a single HBOT session did not increase oxidative damage markers such as MDA. On the contrary, it was accompanied by a reduction in circulating pro-inflammatory cytokines IL-6 and TNF-α [77]. The authors interpreted these findings as evidence of a physiological redox adaptation to transient hyperoxia. This suggests that short-term exposure to HBOT does not cause oxidative injury but rather stabilises the balance between oxidative and antioxidant processes, thereby supporting redox homeostasis under conditions of elevated oxygen availability [77].

Activation of PPARγ, the principal regulator of adipogenesis and insulin sensitivity, promotes adipocyte differentiation and induces the expression of genes characteristic of beige and brown adipose tissue, such as *UCP1* and *PGC-1α*. Previous studies have shown that HBOT modulates PPARγ expression and increases the levels of its endogenous ligands, including 15-deoxy-Δ^12,14^-prostaglandin J_2_ (15d-PGJ_2_) [78]. This may represent an additional mechanism through which HBOT regulates body mass and adipose tissue metabolism.

**Figure 1:**
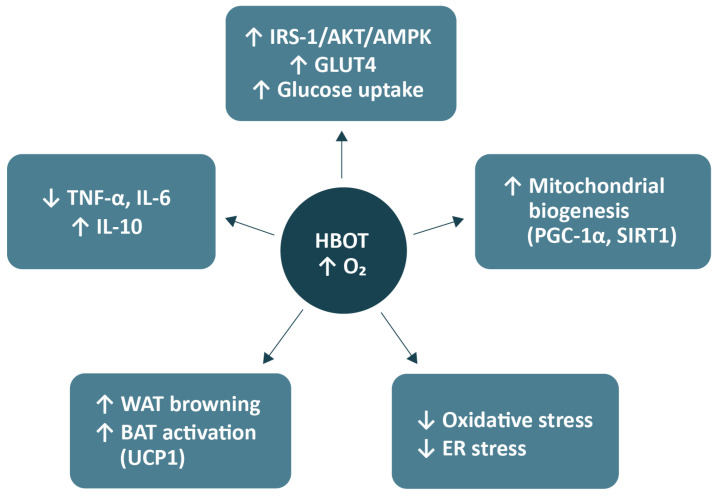
Metabolic mechanisms of HBOT. Abbreviations: HBOT, hyperbaric oxygen therapy; O_2_, oxygen; IRS-1, insulin receptor substrate-1; Akt, protein kinase B; AMPK, AMP-activated protein kinase; GLUT4, glucose transporter type 4; PGC-1α, peroxisome proliferator-activated receptor gamma coactivator 1-alpha; SIRT1, sirtuin 1; ER, endoplasmic reticulum; WAT, white adipose tissue; BAT, brown adipose tissue; UCP1, uncoupling protein 1; TNF-α, tumour necrosis factor-alpha; IL, interleukin. Arrows from the central node (HBOT) indicate downstream effects induced by increased tissue oxygenation. Upward (↑) and downward (↓) symbols within boxes denote relative upregulation or downregulation of the respective pathways or biomarkers. The figure was prepared using the free online software available at https://www.canva.com/.

In the study by Balestra et al. (2022), the effects of repeated hyperoxic exposures (so-called pulsed hyperoxia) were investigated in healthy volunteers, focusing on biomarkers of oxidative stress and mitochondrial biogenesis in peripheral blood mononuclear cells [79]. The results demonstrated that single, short-term exposures to high oxygen concentrations (normobaric hyperoxia) caused a transient increase in advanced oxidation protein products and advanced glycation end-products, indicative of a mild, controlled oxidative stress response [79]. Importantly, this transient oxidative challenge subsequently triggered an adaptive response characterised by increased expression of PGC-1α and TFAM, both key regulators of mitochondrial biogenesis [79]. The authors described this phenomenon as the ‘normobaric oxygen paradox’, in which alternating periods of hyperoxia and normoxia stimulate adaptive mechanisms similar to those induced by physical exercise [66]. The concurrent rise in the expression of PGC-1α and NRF2 in peripheral blood mononuclear cells reflects activation of mitochondrial compensatory and antioxidant pathways, ultimately enhancing oxidative capacity and cellular resistance to oxidative stress [79]. A schematic summary of the proposed mechanisms of HBOT action is presented in Figure 1.

### 3.5. Effects of HBOT on the Gut Microbiome

An increasing body of evidence indicates a close association between obesity, insulin resistance, and the gut microbiota [80]. Although clinical studies on HBOT in the context of obesity remain limited, emerging data suggest its potential to modulate the gut microbiome, which serves as a key regulator of both metabolism and inflammation. The underlying mechanism primarily involves alterations in the local intestinal microenvironment [81]. Elevated partial oxygen pressure in tissues enhances mucosal oxygenation and transiently modifies the intestinal oxygen gradient [81]. This shift favours the restoration of probiotic bacterial populations such as *Bifidobacterium* spp. and *Akkermansia* spp., while suppressing the growth of inflammation-associated microorganisms including *Escherichia coli* and *Enterobacteriaceae* spp. Consequently, HBOT may contribute to the partial correction of dysbiosis and the re-establishment of microbial equilibrium [81,82]. The effects of HBOT on the gut microbiota have also been demonstrated in patients with Crohn’s disease [81,83]. HBOT administration resulted in a significant reduction in inflammatory markers such as interferon-γ, IL-12, IL-17, and TNF-α, along with clinical improvement measured by the Crohn’s Disease Activity Index [81,83]. Changes were also observed in microbial metabolites. Following HBOT, there was an increase in short-chain fatty acids such as butyrate and propionate, which promote epithelial regeneration, exert anti-inflammatory effects, and modulate systemic energy metabolism [84]. In parallel, beneficial alterations occurred in bile acid and sphingolipid metabolism [84].

Through its effects on the gut microbiome, HBOT may play an important role not only in intestinal inflammatory diseases but also in disorders of the central nervous system. In an experimental traumatic brain injury model in rats, HBOT modified gut microbial composition, restoring post-injury reductions in *Prevotella copri* and *Deinococcus* spp. and adjusting the ratio of aerobic to anaerobic bacteria [80]. Importantly, *P. copri* abundance was found to negatively correlate with neuroinflammation severity, brain lesion volume, and activation of the NOD and proteasome signalling pathways—both of which play crucial roles in the progression of inflammatory and neurodegenerative processes [80]. These findings suggest that HBOT-induced modulation of the gut microbiota may represent a novel neuroprotective mechanism, attenuating inflammatory responses along the gut–brain axis [80]. These observations were further supported by another mouse study of traumatic brain injury, where HBOT improved intestinal function, reduced histopathological damage, and lowered inflammatory markers (HIF-1α and aquaporin-4) and tissue oedema [85]. Notably, HBOT increased overall microbial diversity and enhanced probiotic colonisation, particularly of *Bifidobacterium* spp.—a hallmark of a beneficial gut flora [85].

**Figure 2:**
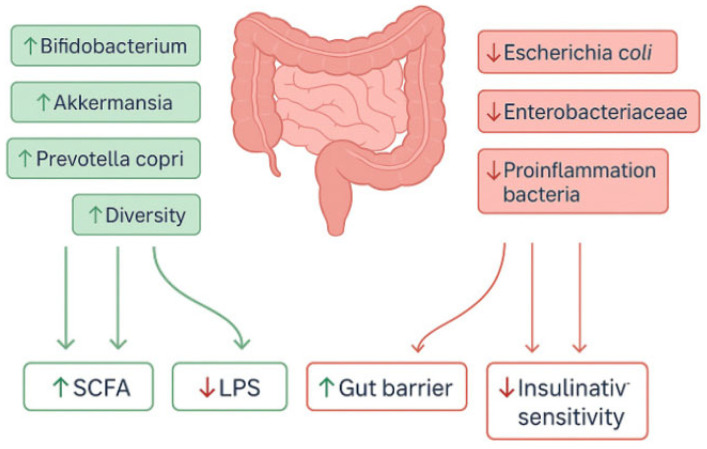
Gut microbiome modulation by HBOT. Abbreviations: HBOT, hyperbaric oxygen therapy; SCFA, short-chain fatty acid; LPS, lipopolysaccharide. Upward (↑) and downward (↓) symbols within boxes denote relative upregulation or downregulation of the respective pathways or biomarkers. The figure was prepared using the free online software available at https://www.canva.com/.

Thus, by modulating pathways involved in fatty acid metabolism and inflammatory regulation, HBOT appears to act through a complex restructuring of the gut–systemic axis [86]. However, some reports indicate that the effects of HBOT on the microbiome may not always be unequivocally beneficial. In one animal model, HBOT increased susceptibility to Clostridioides difficile infection, which the authors attributed to alterations in the aerobic–anaerobic bacterial ratio [87]. Together, these findings highlight that HBOT constitutes a potent tool for microbiome modulation, although the outcomes of such interventions depend strongly on the clinical context and the baseline microbial balance. A schematic representation of the impact of HBOT on gut microbiota composition is shown in Figure 2.

## 4. Evidence from Preclinical and Clinical Studies

### 4.1. Preclinical Studies: In Vivo and In Vitro Findings

Clinical data regarding HBOT in metabolic disorders remain limited. Therefore, a series of preclinical investigations, conducted both in animal models and cell-based systems, have provided valuable insights into the mechanisms by which HBOT may modulate metabolism. The most commonly employed models include rodents (both rats and mice) fed an HFD, animals with streptozotocin-induced diabetes, or genetically obese rats [19,23,24,61]. HBOT protocols typically involve 100% oxygen administered under pressures ranging from 1.5 to 2.5 ATA for periods extending from several days to several weeks, allowing assessment of both acute and chronic effects [23,24,58].

In the study by Liu et al. [23], a murine model of T2DM was used in which obesity was induced by an HFD, and pancreatic β-cell dysfunction was aggravated by streptozotocin administration. Animals underwent HBOT according to a standardised protocol. The therapy led to reduced fasting glycaemia, improved glucose tolerance, and increased insulin sensitivity [23]. Interestingly, HBOT-treated mice also exhibited reduced food intake, suggesting a potential influence of the therapy on appetite-regulating mechanisms. Molecular analysis revealed that HBOT affects several key metabolic pathways. In skeletal muscle, phosphorylation of Akt and AMPK was increased, facilitating GLUT4 translocation and enhancing glucose uptake [23]. In BAT, expression of UCP1 was upregulated, suggesting enhanced thermogenesis and elevated energy expenditure [23]. Concurrently, hypothalamic analysis showed reduced activity of neuropeptide Y neurons, known appetite promoters, potentially explaining the observed reduction in food consumption [23]. In the pancreas, β-cell mass increased, accompanied by decreased islet apoptosis, as indicated by elevated B-cell lymphoma 2 (Bcl-2) expression and reduced caspase-3 activity [23]. Moreover, hepatic glycogen content increased, while gluconeogenic activity decreased. Collectively, these findings indicate that HBOT not only improves peripheral glucose utilisation but also directly protects pancreatic β-cells from injury and supports their regeneration [23]. The findings of Liu et al. [23] were complemented by those of Lee et al. [24], who applied HBOT to diet-induced obese rats at pressures ranging from 1.5 to 2.5 ATA (100% O_2_, 60 min/day for 7 days). The therapy resulted in a significant increase in BAT volume [24]. At the molecular level, increased expression of UCP1 and PGC-1α—key regulators of mitochondrial thermogenesis—was observed, suggesting that HBOT activates BAT and enhances overall energy expenditure [24]. Together, these studies indicate that the metabolic mechanisms of HBOT involve not only improved glucose uptake and utilisation but also activation of thermogenic pathways, mitochondrial stimulation, and enhanced β-oxidation of fatty acids in metabolically active tissues [23,24].

Yuan et al. [61] conducted studies on C57BL/6 mice with HFD-induced obesity, which underwent a 4-week HBOT regimen. The treatment significantly reduced body weight and total adipose mass [61]. The authors reported decreased free fatty acid levels and normalisation of dyslipidaemia parameters. In skeletal muscle, PPARα activity was restored, while CPT1B expression decreased, indicating a balanced rate of fatty acid β-oxidation [61]. In adipose tissue, increased hormone-sensitive lipase activity was observed, promoting lipolysis and mobilisation of stored lipids. These findings suggest that HBOT may modulate metabolism through regulation of L-carnitine levels and activation of the PPARα–CPT1B axis, thereby restoring efficient β-oxidation under conditions of disturbed energy homeostasis [61]. Equally noteworthy are the results of Losada et al., who assessed HBOT as a protective strategy in a rat model of hepatic ischemia. Three HBOT sessions (2 ATA, 60 min) administered before induction of liver injury significantly reduced hepatocyte necrosis and apoptosis and mitigated oxidative stress, as evidenced by lower MDA levels and increased activities of the antioxidant enzymes superoxide dismutase and catalase [88]. Importantly, HBOT led to upregulation of PPARα and PGC-1α—key regulators of mitochondrial biogenesis and fatty acid β-oxidation [88]. The authors proposed that the protective effects of HBOT were mediated not only by prevention of oxidative damage but also by induction of adaptive metabolic pathways that enhance cellular resilience to reperfusion stress [88]. The upregulation of PPARα and PGC-1α, together with improved redox balance, supports the concept that HBOT functions as a form of metabolic ‘oxygen training’, conditioning cells to utilise oxygen and fatty acids more efficiently under oxidative load [88].

Liang et al. [89] focused on a rat model of hypertriglyceridaemia induced by lipid emulsion administration. Animals were exposed to HBOT under two regimens: 2.0 ATA for either 180 or 360 min per day. The shorter exposure (3 h) resulted in a marked reduction in cholesterol and triglyceride levels and exerted cardioprotective effects without evidence of toxicity [89]. Extending the therapy to 6 h amplified the hypolipidaemic effect but simultaneously led to hepatic injury, manifested by elevated levels of alanine aminotransferase and aspartate aminotransferase [89]. This study carries considerable practical significance because it demonstrates a clear dose–response relationship in HBOT and underscores the necessity of optimising exposure protocols to maximise metabolic benefits while minimising adverse effects. Particularly noteworthy are studies investigating HBOT in metabolic liver disease. Chen et al. [84] employed a murine model of metabolic dysfunction-associated steatohepatitis, induced by a combination of a high-fat, high-cholesterol, and methionine–choline-deficient diet. HBOT significantly reduced hepatic steatosis, inflammation, and fibrosis, and improved insulin sensitivity. Importantly, the therapy was associated with pronounced alterations in gut microbiota composition—specifically, an increase in bacterial diversity and enrichment of *Akkermansia* spp., a genus linked to improved metabolic homeostasis [84]. When the gut microbiota was experimentally depleted, the beneficial effects of HBOT were abolished, clearly confirming the involvement of the gut–liver axis in the observed outcomes [84].

In another study, Shwe et al. [90] investigated an accelerated ageing model in Wistar rats induced by chronic administration of D-galactose combined with an HFD. HBOT treatment resulted in significant improvements in cognitive performance and insulin sensitivity. The molecular mechanisms included reduced oxidative stress within the hippocampus, decreased microglial activation and neuronal apoptosis, increased synaptic density, and enhanced mitochondrial biogenesis. These results suggest that HBOT can counteract both metabolic and neurodegenerative dysfunctions associated with ageing [90].

Further evidence was provided by Norouzirad et al. [58], who examined the effects of hyperoxia on metabolism in a rat model of obesity and T2DM. Animals were exposed to elevated oxygen concentrations, after which glucose metabolism, lipid profile, and adipose tissue phenotype were analysed. The study demonstrated a significant improvement in glucose tolerance and insulin sensitivity in hyperoxia-exposed animals. At the tissue level, pronounced browning of WAT was observed [58]. In the interscapular fat depot, expression of UCP1 was markedly increased, along with elevated levels of the transcriptional regulators PPARγ and PGC-1α, both key modulators of thermogenesis [58]. The authors emphasised that these phenotypic transformations in adipocytes were closely correlated with improved carbohydrate metabolism, suggesting that hyperoxia facilitates metabolic reprogramming towards greater energy expenditure and enhanced glucose homeostasis. This study provides important preclinical evidence that modulation of adipose tissue metabolism through increased oxygen availability may represent a promising therapeutic strategy in the management of obesity and T2DM [58]. All key preclinical studies are summarised in Table 1.

### 4.2. Clinical Studies and Trials

Over the past decade, only a limited number of clinical studies have investigated HBOT in patients; however, the available evidence indicates that HBOT may beneficially modulate metabolic parameters in individuals with overweight, obesity, and T2DM. Although these studies often involve small cohorts, they provide meaningful evidence suggesting favourable effects of HBOT on glucose–insulin homeostasis, lipid metabolism, and inflammatory status.

One of the earliest clinical demonstrations of HBOT’s beneficial impact on glucose metabolism was reported by Wilkinson et al. (2015), who studied overweight men, both with and without T2DM [91]. Eight participants underwent five consecutive HBOT sessions, and insulin sensitivity was assessed using the hyperinsulinemia–euglycemic clamp technique. After the treatment series, a significant increase in insulin sensitivity was observed, reflected by a 25–30% elevation in peripheral glucose uptake compared with baseline values [91]. Notably, this effect occurred in both diabetic and non-diabetic participants, suggesting that the improvement in insulin responsiveness following HBOT is not restricted to individuals with manifest diabetes but may also extend to overweight subjects at elevated risk of developing insulin resistance and T2DM [91]. Further evidence was provided by Wilkinson et al. (2020) in a randomised controlled trial involving 25 overweight or obese men with T2DM [20]. Participants were randomly assigned to either an HBOT group or a hyperbaric air control group breathing compressed air under identical conditions. Insulin sensitivity was evaluated via the hyperinsulinemia–euglycemic clamp both during and immediately after exposure. The results showed that in the HBOT group, the glucose infusion rate increased by an average of 26% during exposure and by 23% post-treatment—both statistically significant changes—while no alterations were observed in the hyperbaric air group [20]. Comparative analysis confirmed that the improvement in insulin sensitivity was specifically attributable to oxygen administration under hyperbaric conditions rather than to the hyperbaric environment itself [20].

Additional clinical insights were provided by Sarabhai et al. [10], who employed a comprehensive experimental design incorporating muscle and adipose tissue biopsies and magnetic resonance spectroscopy. In a cohort of 12 patients with T2DM, a single HBOT session reduced fasting plasma glucose by 19%, increased total insulin sensitivity by 34%, and improved mitochondrial bioenergetics in both skeletal muscle and adipose tissue compared with controls [10]. Furthermore, ER stress markers in skeletal muscle were reduced [10]. In another study, Hachmo et al. [92] investigated the effects of long-term HBOT in healthy older adults. The intervention not only improved metabolic parameters but also exerted anti-ageing effects: telomere length in lymphocytes increased by more than 20%, while the proportion of senescent T lymphocytes declined by approximately 10–37%. These molecular changes were accompanied by improvements in cognitive performance, suggesting a link between HBOT, metabolic efficiency, and enhanced resistance to oxidative stress during ageing [92]. It is also noteworthy that the beneficial metabolic effects of HBOT are complemented by observations related to oxidative stress and inflammation. In studies involving healthy volunteers, repeated HBOT sessions were shown to decrease ROS generation in neutrophils without exacerbating systemic oxidative stress or lipid peroxidation [77]. These findings indicate that the intervention is not only effective but also safe with regard to maintaining oxidative and inflammatory balance, further reinforcing its therapeutic potential [77].

All key parameters and outcomes of the discussed studies are summarised in Table 2.

## 5. Safety of HBOT

Given the paramount importance of safety, we considered the potential use of HBOT in special populations such as children, pregnant women, and older adults. To date, there are no clinical trials evaluating HBOT for the treatment of obesity in these groups. Therefore, current considerations rely on animal experiments or on studies of HBOT conducted for other indications.

### 5.1. Paediatric Population

The use of HBOT in children is well documented for several established indications, such as CO poisoning and necrotising soft tissue infections; however, its application in childhood obesity remains unexplored [46]. To date, no clinical studies have specifically investigated HBOT in children in the context of weight reduction or the metabolic syndrome. Nevertheless, several important inferences can be drawn from indirect evidence and safety studies.

A recent report examined a combined intervention involving HBOT, antioxidant therapy, and psychotherapy in a cohort of children aged 10–14 years with obesity and anxiety disorders [47]. In this group, the addition of HBOT sessions (1.6 ATA for 30–45 min, approximately 10 sessions) resulted in a greater reduction in body weight than did lifestyle interventions alone [47]. The children also exhibited psychological improvements, including reduced anxiety, better sleep quality, and, notably, immunological changes such as increased serum immunoglobulin G and immunoglobulin M levels, accompanied by decreased circulating immune complexes [47]. Although preliminary, these findings suggest that HBOT may provide multidimensional benefits for adolescents with obesity, potentially addressing both metabolic and psychological comorbidities.

Safety remains a paramount concern in paediatric HBOT, and the available evidence is largely reassuring. A large retrospective study involving 329 non-obese paediatric patients undergoing HBOT reported a low incidence of complications and no long-term adverse effects [46]. With appropriate precautions—such as pressure equalisation techniques and careful dose control—children tolerated HBOT comparably to adults [46]. The most frequent issues were middle ear barotrauma and anxiety or claustrophobia in younger participants, both manageable with standard procedures. Importantly, no cases of oxygen toxicity involving the central nervous system or lungs were reported. These data indicate that from a safety standpoint, HBOT can be administered to paediatric patients, including those with obesity, provided that strict clinical protocols are observed.

Certain developmental considerations must, however, be taken into account. Children’s organs are still maturing, and the safety of HBOT during prenatal and early postnatal development remains an open question. Experimental evidence suggests potential risks associated with chronic hyperoxic exposure during critical developmental periods [93,94]. Animal studies have shown that excessive oxygen concentrations can impair angiogenesis and promote pathological vascular remodelling, particularly within the retina, thereby increasing the risk of retinopathy of prematurity [95]. Similarly, experiments in neonatal rodents indicate that prolonged hyperoxia may disrupt lung development, leading to lesions resembling bronchopulmonary dysplasia [96]. Preclinical research also demonstrates that sustained exposure to high oxygen levels during the neonatal period may adversely affect brain development [97]. In murine models, hyperoxia impaired neuronal and oligodendrocyte maturation and triggered prolonged neuroinflammatory responses [97]. Histological analyses revealed abnormal myelination and reduced oligodendrocyte differentiation, resulting in deficits in white matter structure and function [97]. Chronic microglial activation and elevated pro-inflammatory cytokine expression persisted into adolescence, suggesting long-term consequences manifesting as neurodevelopmental impairments [97]. Comparable findings were reported by Lithopoulos et al. [93], who found that neonatal mice exposed to high oxygen levels during the first days of life developed persistent cognitive deficits associated with cerebrovascular dysfunction and reduced hippocampal neurogenesis. These observations emphasise the need for caution when considering HBOT during prenatal and early infancy periods: although the therapy offers numerous clinical benefits, inappropriate use could potentially interfere with the growth and maturation of vital organs. Therefore, the application of HBOT in children for non-urgent indications such as obesity should be approached with strong justification and ideally supported by controlled clinical trials. In clinical practice, interventions for paediatric obesity currently focus on dietary modification, physical activity, and behavioural therapy, with pharmacotherapy—such as metformin or glucagon-like peptide-1 receptor agonists—reserved for selected cases [98,99,100,101]. HBOT is not included in any existing clinical guidelines for paediatric obesity. However, should future research confirm its safety and metabolic efficacy in adolescents, HBOT could potentially be considered as part of comprehensive care, particularly in patients with coexisting metabolic or psychological disorders.

### 5.2. Pregnant Women

Pregnancy is generally considered a contraindication for elective HBOT because of concerns about potential risks to the developing foetus [102]. Clinical data on HBOT during pregnancy have produced mixed neonatal and foetal outcomes, reinforcing a precautionary approach. Multiple animal models demonstrate that early hyperoxic exposure can induce adverse developmental programming in the retina, lungs, brain, cardiovascular system, and kidneys [95,96,97,103]. Consequently, HBOT during pregnancy is almost exclusively justified in emergency settings, most notably as a life-saving intervention for CO poisoning, where both the mother and foetus are at high risk if left untreated. In such cases, HBOT is indicated as an emergency therapy and has been shown to prevent maternal and foetal death [102,104]. Although isolated reports have described adverse foetal outcomes—such as miscarriage, preterm delivery, or congenital anomalies—these effects are attributed primarily to CO toxicity itself rather than to HBOT exposure [102]. Current medical consensus holds that HBOT mitigates foetal injury by facilitating rapid elimination of CO and restoring oxygen transport; therefore, treatment of the mother is prioritised, as maternal stabilisation directly benefits the foetus.

By contrast, the use of HBOT for non-emergency conditions such as obesity or gestational diabetes mellitus is not recommended and remains essentially unstudied. Theoretical risks include foetal oxidative stress, as elevated oxygen tensions may impair uteroplacental blood flow or generate ROS beyond the antioxidant capacity of the developing foetus [105]. Additionally, animal studies suggest a potential teratogenic risk, whereby prolonged hyperoxia may restrict foetal growth or induce structural malformations [105]. For example, experiments in pregnant rats have shown that exposure to oxygen at 2–3 ATA for extended durations can result in reduced birth weight and developmental abnormalities in offspring [105]. These findings highlight that, even when HBOT is well tolerated by the mother, the foetus may be adversely affected—particularly during early gestation, when organogenesis is most vulnerable.

**Figure 3:**
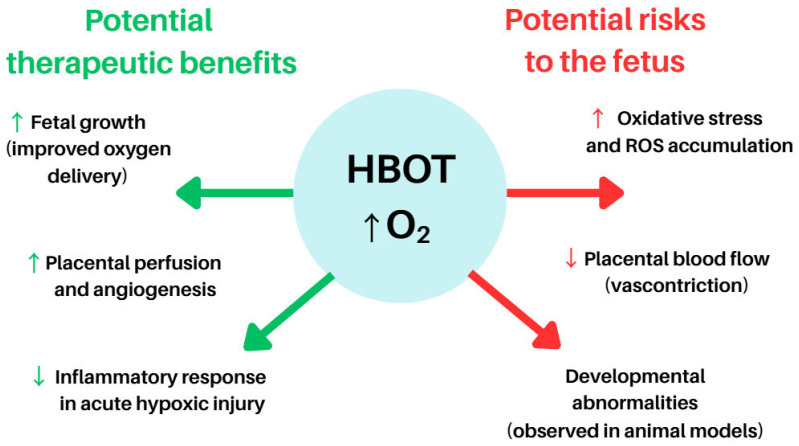
Potential indications for and benefits of HBOT in pregnancy. Abbreviations: HBOT—Hyperbaric Oxygen Therapy, ROS—Reactive Oxygen Species, O_2_—Oxygen, ↑ (up arrow)—indicates an increase, activation, or enhancement of a process, ↓ (down arrow)—indicates a decrease, inhibition, or reduction of a process. The figure was prepared using the free online software available at https://www.canva.com/.

Figure 3 illustrates the potential maternal and foetal effects of HBOT during pregnancy, highlighting both its possible therapeutic benefits and associated risks. While HBOT has been explored in isolated clinical scenarios such as intrauterine growth restriction, placental dysfunction, and decompression sickness during pregnancy, its purported benefits include improved foetal growth, restoration of placental function, and enhanced neuroprotection under controlled hyperoxic conditions [106].

### 5.3. Elderly Patients

Older adults frequently present with coexisting metabolic disorders and may potentially benefit from the regenerative and metabolic effects of HBOT [107]. Indeed, HBOT has been explored as a tool for promoting ‘healthy ageing’. Ageing is accompanied by progressive telomere shortening, cellular senescence, and declines in both cognitive and physical function [92]. Moreover, obesity and diabetes are recognised to accelerate ageing processes through chronic inflammation, oxidative stress, and mitochondrial dysfunction [108,109]. To date, studies in healthy elderly individuals have demonstrated that HBOT exerts significant anti-ageing effects at the cellular level [92]. In obese elderly patients, HBOT may confer multiple benefits: it has been reported to improve insulin sensitivity—similar to effects seen in younger cohorts—to enhance tissue oxygen delivery in poorly perfused regions, and to potentially alleviate age-related osteoporosis and sarcopenia [10,107,110].

**Figure 4:**
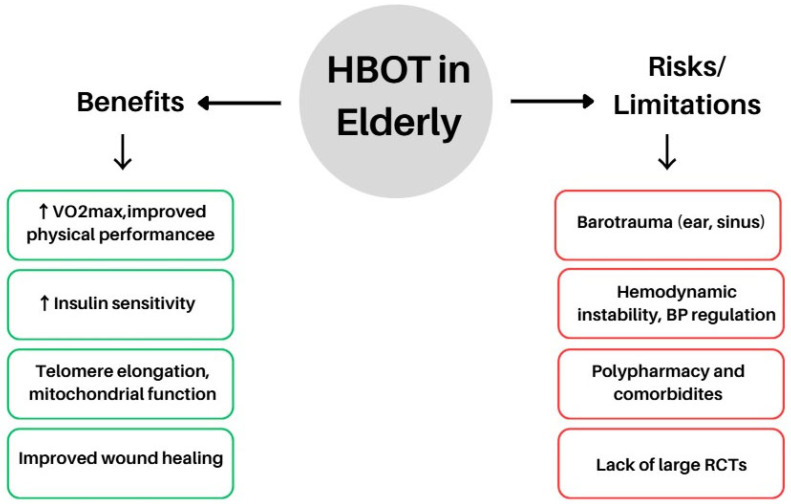
Potential benefits and risks of HBOT in the elderly population. Abbreviations: HBOT, hyperbaric oxygen therapy, VO_2_max, maximal oxygen uptake; BP, blood pressure; RCTs, randomised controlled trials; ↑ (up arrow) → increase, activation, or enhancement of a process; ↓ (down arrow) → decrease, inhibition, or reduction of a process. The figure was prepared using the free online software available at https://www.canva.com/.

A 2022 study by Imerb et al. [110] showed that HBOT improved bone mineral density and microarchitecture in aged rats, including obese specimens, by reducing bone marrow inflammation and osteoclast activity. Similarly, Shwe et al. (2021) observed that in aged, obese rats subjected to D-galactose injections and an HFD, HBOT restored cognitive function and mitigated hippocampal pathology [90]. Notably, severe insulin resistance, a hallmark of ageing and obesity, was significantly improved following HBOT exposure [90]. Clinically, many elderly patients already undergo HBOT for chronic wound management, particularly for diabetic foot ulcers and radiation-induced tissue injuries [111]. Obese individuals often experience impaired wound healing due to microvascular dysfunction and reduced tissue perfusion [112]; in such cases, HBOT can be especially valuable by compensating for local hypoxia. Although these reports are largely anecdotal, clinical observations indicate that elderly obese patients generally tolerate HBOT comparably to younger adults, without a higher incidence of adverse effects—aside from issues such as blood pressure regulation [113] and middle ear barotrauma, which require monitoring in older individuals because of vascular stiffness and Eustachian tube rigidity [114,115]. The principal benefits and potential risks of HBOT in elderly patients are illustrated in Figure 4.

Recent findings suggest that the favourable effects of HBOT in ageing extend beyond vascular and neuroprotective mechanisms to include metabolic regulation [116,117,118]. In a randomised controlled trial involving healthy seniors, HBOT improved cognitive function and cerebral perfusion, reflecting enhanced efficiency in oxygen and energy utilisation within the central nervous system [116]. Similarly, a translational study combining animal models and older patients with features of Alzheimer’s disease demonstrated improved endothelial function and reduced amyloid deposition, likely mediated by modulation of oxidative stress and protein homeostasis [117]. Moreover, in a prospective clinical trial involving patients with cerebral small vessel disease, HBOT combined with folic acid supplementation lowered homocysteine levels—a key biomarker of metabolic dysregulation and vascular risk [118].

Collectively, these findings indicate that even when the primary therapeutic objectives of HBOT are vascular or cognitive restoration, secondary metabolic regulation—both direct and indirect—may play a critical contributory role (Figure 4).

## 6. Perspectives and Conclusions

Current evidence indicates that HBOT holds substantial potential for modulating metabolic processes, insulin resistance, and complications arising from chronic low-grade inflammation. Preclinical studies consistently demonstrate the beneficial effects of HBOT on glucose and lipid metabolism, appetite regulation, BAT activation, mitochondrial function, and gut microbiota composition. Animal models clearly show that HBOT not only enhances insulin sensitivity but also counteracts degenerative and oxidative stress-related processes underlying metabolic ageing. Clinical studies, though limited in sample size, corroborate several of these preclinical findings. In patients with T2DM and obesity, repeated HBOT sessions have been shown to improve insulin sensitivity, reduce glycaemia, and positively influence mitochondrial bioenergetics in target tissues. Moreover, in healthy elderly individuals, HBOT appears to affect fundamental ageing markers such as telomere length and cellular senescence, while simultaneously enhancing cognitive performance and systemic metabolic efficiency. However, the current body of research presents important limitations, including small study populations, short follow-up periods, and heterogeneity in HBOT protocols, making it difficult to draw definitive conclusions regarding the long-term efficacy and safety of this intervention in clinical populations.

Future research should prioritise large-scale, randomised controlled trials to establish optimal therapeutic parameters—such as pressure, duration, and frequency of sessions—and to identify patient subgroups most likely to benefit from HBOT. Equally important will be long-term monitoring of potential adverse effects, which is essential for defining the risk–benefit profile of HBOT as an adjunctive strategy in metabolic and ageing-related disorders.

## Figures and Tables

**Table 1:** Preclinical studies of HBOT in obesity and metabolic disorders (2012–2025). Abbreviations: HFD, high-fat diet; STZ, streptozotocin; T2DM, type 2 diabetes mellitus; ATA, atmospheres absolute; FFA, free fatty acids; BAT, brown adipose tissue; UCP1, uncoupling protein 1; TG, triglycerides; HFHC, high-fat, high-cholesterol; MCD, methionine-choline deficient; MASH, metabolic dysfunction–associated steatohepatitis. Arrows (↑/↓) denote an increase or decrease, respectively, in expression, activity, or physiological parameter compared with control conditions.

Organism	Model (Obesity/Metabolic Inducer)	HBOT Protocol (Pressure, Duration, Frequency)	Key Outcomes	Mechanisms Identified	References
Mice (C57BL/6J)	HFD + STZ diabetic mice (T2DM model)	2.0 ATA, 100% O_2_, 60 min/day for 7 days	↓ Fasting glucose; ↑ insulin sensitivity; ↓ food intake	↑ Muscle p-Akt, p-AMPK, GLUT4; ↑ BAT UCP1; ↓ NPY neuron activity	[23]
Mice (C57/B6)	HFD-induced obese mice	2.0 ATA, 100% O_2_, 60 min/day for 4 weeks	↓ Body weight, ↓ adiposity; ↓ FFA; improved dyslipidaemia	↑ Muscle PPARα; ↓ muscle CPT1B; ↑ adipose HSL; normalised carnitine balance	[61]
Rat (Sprague–Dawley)	Obese rats (diet-induced)	1.5–2.5 ATA, 100% O_2_, 60 min/day for 7 days	↑ Brown fat volume; ↑ BAT glucose uptake; ↓ TG	↑ BAT UCP1 and PGC-1α; ↑ thermogenesis; ↑ energy expenditure	[24]
Mice (C57BL/6J)	STZ-induced T2DM mice (with HFD feeding)	2.0 ATA, 100% O_2_, 60 min/day for 7 days	↓ Fasting glucose; ↑ insulin sensitivity; ↑ β-cell mass	↓ Pancreatic β-cell apoptosis; ↑ liver glycogen;	[19]
Rat (Sprague–Dawley)	Hyperlipidaemic rats (fat emulsion injection)	2.0 ATA, 100% O_2_, 180 min vs. 360 min/day for 14 days	3 h: ↓ cholesterol, ↓ TG, cardioprotection, no toxicity; 6 h: greater lipid reduction but liver injury	HBOT dose–response observed; 6 h caused oxidative liver damage whereas 3 h improved metabolism safely	[89]
Mice (C57BL/6J)	MASH mice (12-week HFHC diet + 4-week MCD diet)	2.2 ATA, 100% O_2_, 60 min/day for 4 weeks	↓ Steatosis, ↓ liver inflammation and fibrosis; ↑ insulin sensitivity	↑ Gut microbiota diversity; shift in liver sphingolipid metabolism; abolished effect when microbiota removed	[84]
Rat (Wistar)	Ageing + obese rats (D-galactose + HFD model)	2.0 ATA, 100% O_2_, ~80 min/day for 14 days (intermittent)	↑ Cognitive function; ↑ insulin sensitivity; ↓ oxidative damage in brain	↓ Hippocampal microglial activation and apoptosis; ↑ synaptic density; ↑ antioxidant enzymes; ↑ mitochondrial biogenesis	[90]
Rat (Wistar)	Rat primary adipocytes (HFD/T2D model)	95% O_2_, 120 min/day for 6 days/5 weeks	↑ Brown/beige adipocyte markers in ‘white’ fat cells	↑ UCP1 expression in adipocytes; ↑ mitochondrial genes	[58]

**Table 2:** Clinical studies of HBOT in metabolic health. Abbreviations: HBOT, hyperbaric oxygen therapy; ATA, atmospheres absolute; MRS, magnetic resonance spectroscopy; HOMA-IR, Homeostatic Model Assessment for Insulin Resistance; WAT, white adipose tissue; ER, endoplasmic reticulum; ROS, reactive oxygen species; MDA, malondialdehyde; ↑ (up arrow) → increase, activation, or improvement; ↓ (down arrow) → decrease, inhibition, or reduction.

Population	Sample Size	HBOT Protocol	Endpoints and Outcomes	Key Findings	References
Men (overweight with/without T2DM)	n = 8	2.8 ATA O_2_, 90 min/day × 5 days	Insulin sensitivity via clamp	↑ Insulin sensitivity	[91]
Men (with T2DM)	n = 25	2.0 ATA O_2_ vs. 2.0 ATA air, 90 min/day	Insulin sensitivity (HOMA-IR, clamp)	Improved clamp insulin uptake; ↓ blood glucose	[20]
Men (with T2DM)	n = 12	2.4 ATA O_2_, 120 min single session	Hyperinsulinemia clamp; muscle and adipose biopsies; MRS	↓ Fasting glucose; ↑ whole-body insulin sensitivity; ↑ muscle and WAT mitochondrial activity; ↓ muscle ER stress	[10]
Healthy older adults	n = 30	2.0 ATA O_2_, 90 min × 60 sessions over 3 months	Telomere length; senescent cell counts; cognition	↑ Telomere length in blood cells; ↓ senescent T cells	[92]
Healthy volunteers	n = 15	2.4 ATA O_2_, 90 min × 5 sessions	Oxidative stress (ROS, MDA); inflammatory markers	↓ ROS production in neutrophils; no increase in MDA; no systemic oxidative stress	[77]

## Data Availability

No new data were created or analysed in this study.

## Abbreviations

The following abbreviations are used in this manuscript:

Akt	Protein kinase B
AMPK	AMP-activated protein kinase
ApoE^−^/^−^	Apolipoprotein E-deficient
ATA	Atmospheres absolute
BAT	Brown adipose tissue
Bcl-2	B-cell lymphoma 2
CO	Carbon monoxide
CPT1/CPT1B	Carnitine palmitoyltransferase 1/1B
DB/db/db	Diabetic (leptin receptor-deficient) mice
ER	Endoplasmic reticulum
FFA	Free fatty acids
GLUT4	Glucose transporter type 4
HBOT	Hyperbaric oxygen therapy
HFD	High-fat diet
HFHC	High-fat, high-cholesterol diet
HIF-1α	Hypoxia-inducible factor 1-alpha
HMOX1	Haem oxygenase 1
HOMA-IR	Homeostatic Model Assessment of Insulin Resistance
HSL	Hormone-sensitive lipase
IL	Interleukin
IRS-1	Insulin receptor substrate 1
LPS	Lipopolysaccharide
MASH	Metabolic dysfunction-associated steatohepatitis
MCD	Methionine–choline-deficient (diet)
MDA	Malondialdehyde
MRS	Magnetic resonance spectroscopy
NF-κB	Nuclear factor kappa-light-chain-enhancer of activated B cells
NLRP3	NOD-like receptor family pyrin domain-containing protein 3
NRF2	Nuclear factor erythroid 2-related factor 2
O_2_	Oxygen
OLETF	Otsuka Long-Evans Tokushima Fatty rat
PGC-1α	Peroxisome proliferator-activated receptor gamma coactivator 1-alpha
PPAR	Peroxisome proliferator-activated receptor
PPARα	Peroxisome proliferator-activated receptor alpha
PPARγ	Peroxisome proliferator-activated receptor gamma
PPARδ (PPARβ/δ)	Peroxisome proliferator-activated receptor delta (beta/delta)
ROS	Reactive oxygen species
SCFAs	Short-chain fatty acids
SIRT1	Sirtuin 1
STZ	Streptozotocin
T2DM	Type 2 diabetes mellitus
TFAM	Mitochondrial transcription factor A
TG	Triglycerides
TNF-α	Tumour necrosis factor-alpha
UCP1	Uncoupling protein 1
VO_2_max	Maximal oxygen uptake
WAT	White adipose tissue
